# Effects of developing scenario learning in a fundamental nursing course: a pilot study

**DOI:** 10.1186/s12909-022-03462-z

**Published:** 2022-05-25

**Authors:** Kyoung-Hwa Baek, Jeong-Hwa Cho, Jongmin Park

**Affiliations:** 1 Department of Nursing, Gyeongbuk College of Health, Gimcheon, Republic of Korea; 2 Department of Nursing, Daejeon Institute of Science and Technology, Daejeon, Republic of Korea; 3 College of Nursing, Research Institute of Nursing Science, Pusan National University, Yangsan, Republic of Korea

**Keywords:** Developing scenario Learning, Nursing student, Team efficacy, Systems thinking, Proactivity in problem-solving

## Abstract

**Background:**

This pilot study aimed to investigate the effects of developing scenario learning (DSL) on team efficacy, systems thinking, and proactivity in problem-solving in a fundamental nursing course.

**Methods:**

A total of 53 second-year nursing students were enrolled in the study; the DSL nursing education program was implemented for 15 weeks from March 4 to June 17, 2021. Data on team efficacy, systems thinking, and proactivity in problem-solving were measured before and after the DSL-based nursing education program. The collected data were analyzed using the IBM SPSS Statistics version 22.0.

**Results:**

The results demonstrated that nursing students’ team efficacy (t = − 8.228, *p* < .001, Cohen’s d = 1.079), systems thinking (t = − 9.757, *p* < .001, Cohen’s d = .731), and proactivity in problem-solving (t = − 8.635, *p* < .001, Cohen’s d = .992) significantly increased after the program.

**Conclusions:**

The findings of this study can contribute to the development of nursing competency in nursing students without experience in nursing practicum. The authors recommend incorporating DSL in the nursing curriculum to promote early adaptation in clinical settings.

## Background

Practical forms of knowledge critical to the core competencies valued in the twenty-first century—problem-solving, language competence, self-directedness, and leadership—are imperative for learners. These help promote success in life and/or advance their professional career [1]. Curricula for nursing students, who must acquire nursing competencies, encompass theoretical and practical field knowledge and are designed to help students build a thought process appropriate for nursing professionals [2]. Education enables nursing students to become competent nurses who plan and administer multi-dimensional and cost-effective care by integrating their knowledge and skills based on social duty and ethics [3]. Although the dramatic advances in the healthcare environment in recent years have bolstered patient safety, emphasis on patients’ rights has decreased the opportunities for nursing students to acquire hands-on experience in patient care and deal with nursing issues in clinical settings [4]. Additionally, the excessive emergence of new nursing schools to meet the growing demand for nurses has led to a shortage of nursing practicum sites, further shifting the hands-on learning paradigm required for practicum to observation-focused learning [5]. Newly-graduate nurses face a transition shock, work-related stress, and perceived inadequacy due to the disparity between their education in school and the competencies demanded by the clinical practice [6]. This, in turn, leads to a high turnover rate and an increasing number of nurses on leave, thereby creating gaps in their nursing careers. Therefore, a nursing curriculum based on program outcomes emphasizes the use of various teaching methods for practice-focused competency training [7], and teaching approaches such as problem-based learning (PBL), simulation, flip learning, and action learning are employed.

Developing scenario learning (DSL) is a learning strategy combining elements of PBL and role-play; it allows students to reflect on various perspectives and interpretations of a scenario by developing the scenario themselves [8]. PBL is a fundamentally static learning strategy that facilitates and nurtures students’ competencies through a structured process with clear-cut problems [9]. Even if a scenario reflecting a real-world clinical situation is implemented, the set framework of the scenario cannot be altered during learning; thus, the focus is purely on an individual’s ability to respond to problems [8, 10]. DSL trains students to respond to environmental changes and reevaluate a piece of information beyond the initial interpretation as the problem at hand evolves. Hence, it facilitates the achievement of learning goals by promoting a problem-solving process that reflects the clinical scenario, critical thinking, confidence, and creativity [9, 11]. Moreover, coupling the key advantage of role plays—performing an unfamiliar role and perspective based on their individual beliefs [12]—with nursing major courses is believed to help nursing students adjust quickly to clinical settings by acquiring core competencies required for the same.

Multidisciplinary approaches are needed to resolve patients’ problems in patient-centered care, with team efficacy being an essential competency demanded of nursing students. Team-learning activities provoke intrinsic motivation, such as team efficacy, and contribute to stimulating the proactive sharing of information among college students [13]. In a meta-analysis of 67 studies, Gully et al. [14] reported that team efficacy boosts confidence among team members and significantly influences work generalization and the outcomes of work. Thus, it is necessary to investigate whether DSL, which involves developing a scenario and resolving problems, is a learning strategy that enhances team efficacy.

Systems thinking refers to the ability to integrate various pieces of knowledge and intuitively view interactions among constructs to learn the entire system effectively [15]. To gain an adequate understanding of system-based patient-centered care to promote the safety and quality of care, nursing students are required to develop an understanding of the overall health management system, as opposed to tackling fragmentary problems affecting individuals [16]. There is no single process for deriving and resolving a problem [8]. Thus, systems thinking has benefits in the complex process of listing various relevant factors when identifying the problem and discussing the various methods to solve the same.

Proactivity in problem-solving refers to recognizing the problem at hand and appropriately responding to any issues that arise while solving the task [17]. As patients in need of care face a disease-specific and multi-dimensional problem situation, nurses are demanded to employ proactive and systematic problem-solving skills. However, an observation-focused nursing practicum is a serious barrier to problem-solving training among nursing students; further, it hinders their use of problem-solving skills in clinical practice following graduation [18]. Therefore, the nursing curricula should expose nursing students to various problem-solving situations and foster a participatory attitude toward problem-solving in the organization. Scenario-based learning has been reported to increase knowledge and/or creative ability for problem-solving by inducing a deep understanding of information [19].

One of the studies that reported the effects of DSL, which also involved the sharing of feedback, examined the effects of emergency patient triage training through a scenario development process and role-play [20]. Further, a hybrid study examined nursing students’ perception of rudeness using DSL [21]; additionally, another study developed a corrective learning program using role-play for nursing students [22]. However, these studies did not adopt a clear definition of DSL and present evidence. DSL has often been studied without being distinguished from learning strategies that combine existing learning methods. Moreover, the strategies were focused on the outcome-centered feedback from the instructor, as opposed to the development of a scenario, thus impairing the understanding of the essence and learning outcomes of DSL.

This study aimed to implement a DSL program for second-year nursing students following the multi-phase protocol outlined by Dalziel [8] and the Core Clinical Nursing Skills Assessment Protocol Version 4.1 (2018), developed by the Korean Accreditation Board of Nursing Education (KABONE). The objective was to assess the effects of DSL on team efficacy, systems thinking, and proactivity in problem-solving to determine the program’s efficacy. The authors believe that DSL can help educate nursing students, enabling them to demonstrate their core clinical competencies in nursing practicum. This will ensure that they quickly adjust to clinical settings as newly graduated nursing professionals embarking on their nursing careers.

This pilot study aimed to develop and apply a DSL strategy in a fundamental nursing course and evaluate the effects of DSL on nursing students’ team efficacy, systems thinking, and proactivity in problem-solving.

## Methods

### Study design

This pilot study employed a one-group pretest-posttest design to investigate the effects of DSL on team efficacy, systems thinking, and proactivity in problem-solving in second-year nursing students.

### Participants

Fifty-three second-year nursing students signed an informed consent form to participate in the study. The sample size was determined using the G*power 3.1.2 program. For the difference from constant (one sample case) with an effect size of 0.5, a significance level of 0.05, and a power of 0.90, the minimum sample size was calculated to be 44. The sample size was determined to be 53, considering a 20% dropout rate. Students who took the 15-week fundamental nursing course from March 4 to June 17, 2021, completed the study questionnaire. There are no incomplete questionnaires; all data from 53 students were included in the final analysis.

### Study procedure

#### Development of the DSL-based nursing education program

To develop the DSL-based fundamental nursing education program, the course syllabus and lesson flow chart were prepared. Thus, the five phases of the DSL model were applied, underpinned by the theoretical basis of core clinical nursing skills and objectives of the fundamental nursing course (Table 1). The DSL model comprises five phases: phase 1—introducing the overall learning experience– sharing of pre-learning; phase 2—preparation of the components–fishbone–identifying the problem, in which students reflect on the scenario through discussions; phase 3—development of a scenario–writing lines, in which students come up with a draft scenario; phase 4—finalization of scenario–application of background, investigation of theoretical evidence, and finalization of the storyboard, in which students reached a consensus on the final scenario; and, phase 5—re-finalization of the scenario–reformulation based on discussion, in which students reviewed and reflected on the interpretations of the initial scenario [9]. The program was designed as a 15-week, 2-credit course (2 hours) in accordance with the curriculum of the school of study. The program was developed by a panel comprising three professors with more than 10 years of clinical nursing experience and 3 years of teaching experience in the fundamental nursing course and three staff assistants with greater than 5 years of clinical nursing experience.

**Table 1:** Procedure and Contents of Developing Scenario Learning-Based Fundamental Nursing Education Program

Theory Phase	Theoretical Sub-process	Curriculum Activities	Supported Technologies
Phase 1 Sharing learning experiences	Introducing the overall learning experience for creating a draft scenario	Identifying learning goals, process and evaluation criteria Sharing prior learning by team	Lecture plan Basic workbook Lecture notes Video clips with online resource
Phase 2 Making components	Create an overview of the scenario by generating queries according to various perspectives	Selection of core basic nursing skills Factors that can occur during skill are set as components	Checklist of nursing skills Fishbone diagram
Phase 3 Creating scenarios	Build hypotheses based on initial scenario interpretation	Convergence of incidents and derived nursing problems Experience the creation of various scenarios	Communication dialog Communication analysis book
Phase 4 Agreeing scenario	Agree on scenario development through a common pattern of discussions	Create a scenario story that reflects the subject’s contextual characteristics with the dialog book selected after discussion Create a scenario module	Scenario module
Phase 5 Reestablish the scenario	Rethinking and reflecting the interpretations of the scenario	After deciding a role with a scenario story, the role-play proceeds Draw, share, and reflect on experiences in the scenario development process	Scenario evaluation tool Reflection log

In the content development stage, four core nursing skills (intermittent gastric tube feeding, intradermal injection, subcutaneous injection, and intramuscular injection) were selected from 20 core nursing skills presented by the KABONE, considering the rate of students’ needs. The assessment protocol for the corresponding core nursing skills was used. Further, online video lectures were recorded, and templates were prepared, for therapeutic communication analysis and skill-centered scenario development report for the program.

#### Implementation of the DSL-based nursing education program

Of the total 15 weeks of fundamental nursing classes, 3 weeks of orientation were conducted. After 4 weeks of lectures by areas, learning activities were conducted to develop scenarios through phases 1 to 4 for 4 weeks. The participants were divided into five teams, and each team comprised seven to eight individuals. In the 13th week, a video was produced while role-playing with the final scenario. In the 14th week, the instructor provided feedback using the learning goal and scenario evaluation tool through a presentation, and the learning activities of phases 4 and 5 were completed. The theme of the DSL-based nursing education program developed by each team is presented in Table 2. An example of the developed finalized scenario is presented in Table 3.

**Table 2:** Theme of the Developing Scenario Learning-Based Nursing Education Program Developed by Each Team

Team	Theme of the Scenario
I	Case of antibiotic skin reaction test by ignoring the history of side effects of antibiotics
II	Case of side effects after antibiotic administration without checking the skin test
III	Case of hypoglycemia due to an error in the insulin dose to be administered
IV	Case where aspiration occurred because the position of the gavage was not confirmed before gavage
V	Case where the purpose of administration and precautions were not explained before intramuscular injection

**Table 3:** Example of Developed Scenario Contents by Developing Scenario Learning

Learning Goals	Developed Scenario content	Scenario Report Evaluation Item
Effective communication before antibiotic skin test	■ (Washing hands. Confirming the patient’s prescription and preparing the item) ■ Nurse: Hello, my name is OOO. (Washing hands) What is your name? ■ Patient: This is △△△. ■ Nurse: (Checking the patient’s bracelet and medication label) 123,456 △△△ has been confirmed. Have you ever taken antibiotics in the past? ■ Patient: Yes. I had a very bad cold and was hospitalized. ■ Nurse: Alright. Have you ever had side effects such as skin rash, itching, heat, and chest tightness after taking antibiotics? ■ Patient: Itchy and red marks around the arm; penicillin or something is not good for me. ■ Nurse: Yes. I know. From now onward, as there is a risk of infection through surgery, antibiotics will be administered. First, to determine if there is a hypersensitivity reaction to antibiotics before administration, we will start with a skin test.	· Hand hygiene · Patient identification · Problem assessment · Select related core skills
Perform the intradermal injection accurately	■ Nurse: (Choose an injection site and take a comfortable position and wash hands) I will do a skin test on your right arm. It will sting slightly (draw the injection site boundary after intradermal injection, and write the date, time, and drug name). ■ Patient: Ah ~ ~ Ah ~ ~ It hurts. ■ Nurse: Were you very sick? I’ll check the skin reaction in 15 minutes. Do not touch or rub the area drawn with the ballpoint pen (Washing hands after organizing).	·Performing core skills · Proceed with the correct procedure
Solve problems through verbal and non-verbal communication	■ Nurse: (After 15 minutes) Show me the area marked with the ballpoint pen (check the degree of redness and swelling). There is a possibility that the test will be positive. Have you ever touched the injection site? Let’s check again. ■ Patient: Why are you doing it again? Isn’t it strange that it’s an antibiotic or something? You said I was allergic. Shouldn’t you find out more before giving an injection? ■ Nurse: Yes. You’re right. As this can happen, I asked you a question before the reaction test, but there is a point after which I am not supposed to check again without consulting the doctor. I’ll check for itching or hives. Are you feeling out of breath? ■ Patient: Did someone else’s injections go wrong? ■ Nurse: You haven’t been on antibiotics yet. As I explained at the beginning, the antibiotic was not administered because the reaction test was performed before the antibiotic injection. So don’t worry too much. We asked you several troublesome questions to protect your safety. Thank you for answering the question, although it is difficult. Do you have any more questions?	· Problem-solving · Determining whether the situation is to be reported to the doctor

In this study, Phase 1 of DSL involved the sharing of initial learning experiences within teams. The students viewed the video lectures, read studies related to core basic nursing skills in advance, and shared their understanding and interpretation of the learning materials with their team during the class. Phase 2 comprised students developing an outline of their scenario through discussion. They chose one core nursing skill and set their skill-focused learning goals and events that may occur while administering the skill accordingly. They used a fishbone diagram to ensure diversity and clarity of nursing problems that may occur while performing the skill. Phase 3 consisted of students developing the draft scenario. They experienced the development of diverse scenarios by writing out several possible cases of conversation scripts and analyses, integrating each event with the identified nursing problems, continuously discussing them with the team, and searching for theoretical evidence. In Phase 4, students reached a consensus on the scenario development. They developed a scenario storyboard using the script chosen by the team members and reflecting the contextual features of the patients involved. In Phase 5, students reviewed the interpretations of the initial scenario. They assigned each other roles based on their team’s storyboard and performed the role-play. They filmed their role-play for presentation and discussion during the class. Further, they wrote a reflection journal to share and reflect on their experiences during scenario development.

### Instruments

#### Team efficacy

The authors used the eight items of the team efficacy subscale used in the study by Marshall and Lori [23] and modified and validated by Kwon [24]; these were rated on a five-point Likert scale. The total score ranges from 8 to 40, and a higher score indicates higher team efficacy. The reliability of the tool was Cronbach’s α = .96 in the study by Kwon [24] and .88 and .95 before and after the intervention, respectively, in this study.

#### Systems thinking

The tool developed by Lee et al. [25] was used. This 20-item tool comprises five domains, with four items each for systems thinking, personal proficiency, mental model, shared vision, and team learning. Each item is rated on a five-point Likert scale. The total score ranges from 20 to 100, and a higher score indicates greater systems thinking. The reliability of the tool was Cronbach’s α = .83 at the time of development of the tool and .81 and .86 before and after the intervention, respectively, in this study.

#### Proactivity in problem-solving

From the five-factor scale for team skills developed by Marshall and Lori [23], the authors used the eight items of the adaptability factor adapted by Kwon [24]. Each item is rated on a five-point Likert scale. The total score ranges from 8 to 40, and a higher score indicates greater proactivity in problem-solving. The reliability of the tool was Cronbach’s α = .77 in the study by Kwon [24] and .77 and .92 before and after the intervention, respectively, in this study.

#### Data analysis

The collected data were analyzed using the IBM SPSS Statistics version 22.0 (SPSS New York, USA). The participants’ demographic characteristics were analyzed with the real number and percentage of mean and standard deviation. Further, the changes in team efficacy, systems thinking, and proactivity in problem-solving after the implementation of the DSL nursing education program were analyzed with paired t-tests. To investigate effect sizes (Cohen’s d), the authors calculated the mean difference of outcomes between the pre- and post-intervention.

## Results

### Demographic characteristics

The participants’ mean age was 19.90 ± 1.95 years. The sample comprised 77.4% female and 22.6% male nurses. The participants’ reasons for choosing their major were as follows: employment (58.5%), recommendation by others (24.5%), based on one’s own decision (15.1%), and based on entrance exam score (1.9%). Adaptation to the nursing major was moderate (81.1%), followed by good (15.1%) and bad (3.8%). Further, the satisfaction with the nursing major was as follows: satisfied (49.1%), moderate (49.1%), and dissatisfied (1.8%). The participants’ GPA until the preceding semester was 3.0–3.9 (71.7%), followed by < 3.0 (17.0%) and ≥ 4.0 (11.3%) (Table 4).

**Table 4:** Demographic Characteristics of the Participants (*N* = 53)

Characteristics	Categories	N (%) or M ± SD
Age (years)		19.90 ± 1.95
Gender	Female	41 (77.4)
	Male	12 (22.6)
Religion	Yes	18 (34.0)
	No	35 (66.0)
Motivation for admission	Self-select	8 (15.1)
	Recommendation of others	13 (24.5)
	Employment	31 (58.5)
	According to grades	1 (1.9)
Adaptation of nursing	Good	8 (15.1)
	Moderate	43 (81.1)
	Bad	2 (3.8)
Nursing satisfaction	Satisfaction	26 (49.1)
	Usually	26 (49.1)
	Dissatisfaction	1 (1.8)
School grades	≥ 4.0	6 (11.3)
	3.9 – 3.0	38 (71.7)
	< 3.0	9 (17.0)

*Note*: *M* mean, *SD* standard deviation

### Effect of the DSL-based nursing education program

The mean team efficacy score significantly increased statistically from 3.70 ± 0.46 before the intervention to 4.23 ± 0.52 after the intervention (t = − 8.228, *p* < .001, Cohen’s d = 1.079). The mean systems thinking score significantly increased statistically from 3.50 ± 0.36 before the intervention to 3.79 ± 0.43 after the intervention (t = − 9.757, *p* < .001, Cohen’s d = .731). Regarding the subscales of systems thinking, the mean scores significantly increased statistically for systems thinking (t = − 5.791, *p* < .001, Cohen’s d = .631), mental model (t = − 4.539, *p* < .001, Cohen’s d = .630), shared vision (t = − 6.180, *p* < .001, Cohen’s d = .504), and team learning (t = − 3.811, *p* < .001, Cohen’s d = .479) after the intervention. However, the mean scores for personal mastery (t = − 0.678, *p* = .501, Cohen’s d = .060) did not change significantly (Table 5). Further, the mean proactivity problem-solving score significantly increased statistically from 3.56 ± 0.41 before the intervention to 4.07 ± 0.60 after the intervention (t = − 8.635, *p* < .001, Cohen’s d = .992) (Table 5).

**Table 5:** Effect of Developing Scenario Learning-Based Fundamental Nursing Education Program (*N* = 53)

Variables	Categories	Pre	Post	t	*p*	Cohen’s d
		M ± SD	M ± SD			
Team efficacy		3.70 ± .46	4.23 ± .52	−8.228	<.001	1.079
Systems thinking		3.50 ± .36	3.79 ± .43	−9.757	<.001	.731
	Systems thinking	3.23 ± .62	3.70 ± .85	−5.791	<.001	.631
	Personal mastery	3.99 ± .51	4.02 ± .48	−.678	.501	.060
	Mental model	3.00 ± .54	3.37 ± .63	−4.539	<.001	.630
	Shared vision	3.41 ± .49	4.02 ± 1.64	−6.180	<.001	.504
	Team learning	3.87 ± .48	4.13 ± .60	−3.811	<.001	.479
Proactivity in problem-solving		3.56 ± .41	4.07 ± .60	−8.635	<.001	.992

*Note*: *M* mean, *SD* standard deviation

## Discussion

This pilot study aimed to develop, implement, and evaluate the effects of the DSL in a fundamental nursing course on team efficacy, systems thinking, and proactivity in problem-solving in nursing students. The participants’ team efficacy score increased from 3.70 before the program to 4.23 after the program. This is consistent with a previous study’s findings that demonstrated an improvement of team efficacy after team-based learning, such as simulation education and blended learning [26, 27]. Team efficacy refers to team members’ belief that their team can successfully undertake a particular task; team members are influenced by other members’ beliefs, faith, and behavior as they coordinate their own behaviors [14]. It is believed that the DSL introduced in the course naturally increases team efficacy by making the group actively create scenarios and perform the role-playing process with various perspectives.

The participants’ systems thinking improved from 3.50 before receiving the program to 3.79 after the program. These results were similar to those of Cho and Hwang [28], who studied nursing students by utilizing the same tool and obtained lower scores than those of Im and Lee [29], who applied a writing program to improve systems thinking. Systems thinking is a useful method to analyze the whole without being constrained by the cross-sectional area, understand the interactions of various factors when making decisions, and solve problems [30]. In this study, it is believed that the systems thinking ability was expressed while analyzing cyclical scenario questions, discussion process, and dialog book according to the step-by-step procedure of the theoretical framework and while writing a reflection journal. Further, in this study, personal proficiency, mental model, and shared vision—the subscales of systems thinking—significantly improved after the program. Systems thinking is the ability to think with the attributes of a dynamic system, a holistic view, and a transformational aspect [29, 31]. DSL involves analysis and transformational thinking for a wide range of environments through self-change and constant interaction with the outside world in the process of moving forward. Moazez et al. [32] investigated nurses’ perceptions of nursing stability and systems thinking. They stated that systems thinking was an influential factor in enhancing nurses’ stability and quality; in terms of healthcare, it has been studied as a major competency in preventing infection control and negative health-related outcomes [33]. Thus, systems thinking will be established as an essential competency in dealing with nursing problems composed of complex causal relationships and improving the nursing process.

Proactivity in problem-solving in nursing students improved to 3.56, which further improved to 4.07 after the program was provided. These results are inconsistent with the study of Byeon [34], which indicated no significant change in proactivity in problem-solving before and after the simulation-based integrated practicum. However, in their study, proactivity in problem-solving improved to a level similar to that in previous studies using the same tools for the same subject group [17, 28, 35], which is consistent with the results of this study. Problem-solving is a cognitive and behavioral process that requires a high-level thinking process. It provides a series of practical solutions to accumulate effective decision-making processes and select the most appropriate solution [36]. The approach to problem-solving is an essential factor for nursing students educated to become professional nurses. This is because rational and reasonable thinking, creativity, intuition, and imagination are introduced to enable clinical judgment or reasoning on nursing problems. DSL approaches are believed to have raised the level of proactivity in problem-solving of the study participants by providing opportunities to collaborate through communication and coordination with members and actively participate in problem-solving [37] in the curriculum.

This study’s findings identified that DSL is effective in team efficacy, systems thinking, and proactivity in problem-solving in nursing students. Considering that DSL was effective for lower-grade students without clinical experience, it is necessary to repeatedly study it as a learning method to help nursing students adapt early to the clinical settings.

## Conclusions

This pilot study provides initial evidence that a DSL-based nursing education program in a fundamental nursing course improves nursing students’ team efficacy, systems thinking, and proactivity in problem-solving. However, this study has limitations in generalizing the results because the convenience sampling method was used to recruit nursing students from one university, and the control group was not assigned. Based on the above results, the following recommendations are made. First, it is necessary to increase the accuracy of the DSL effect by assigning experimental and control groups and controlling exogenous variables due to the educational environment. Second, it is necessary to introduce DSL methods into various subjects in addition to the fundamental nursing education program and develop and apply modules.

## Data Availability

The datasets generated and/or analyzed during this study are not publicly available due to the datasets containing information that could compromise research participant consent. However, the datasets are available from the corresponding author upon reasonable request.

## Abbreviations

DSL: Developing Scenario Learning
PBL: Problem-Based Learning
KABONE: Korean Accreditation Board of Nursing Education

## References

[1] Tick A. Application of problem-based learning in classroom activities and multimedia. In: 5th Slovakian hung joint symposium on applied machine intelligence and informatics. Poprad; 2007. p. 363–75. Retrived from http://bmf.hu/conferences/sami2007/36_Andrea.pdf.

[2] Nehrir B, Vanaki Z, Mokhtari Nouri J, Khademolhosseini SM, Ebadi A (2016). Competency in nursing students: a systematic review. Int J Travel Med Glob Health.

[3] American Association of Colleges of Nursing. The essentials: Core competencies for professional nursing education. Unpublished draft November 2020. p. 5.

[4] Kim E, Kang S (2016). Effects of the simulation on the Ego resiliency, self-efficacy and satisfaction of major of the nursing students. J Korea Acad Ind Coop Soc.

[5] Chung MS, Park JS, Ryu E, Shin G, Jun HY, Kim BJ (2015). Teaching effectiveness and adequacy of practical training in nursing students. J Korea Acad Soc Nurse Educ.

[6] Jung D, Lee SH, Kang SJ, Kim JH (2017). Development and evaluation of a clinical simulation for new graduate nurses: a multi-site pilot study. Nurse Educ Today.

[7] Kim YM, Park H. Current trends of teaching-learning methods in Korean undergraduate nursing education. J Learn Cent Curric Instr. 2016. 10.22251/jlcci.2016.16.11.945.

[8] Dalziel J, Cameron L, Dalziel J (2012). Developing Scenario Learning and its implementation in LAMS. Proceedings of the 7th International LAMS conference: surveying the learning design landscape.

[9] Chan ZC (2012). Role-playing in the problem-based learning class. Nurse Educ Pract.

[10] Cameron L, Abas ZW, Jung I, Luca J (2010). Why re-invent the wheel? Sharing teaching strategies that work. Proceedings of the Global Learn Asia Pacific.

[11] Olaussen C, Heggdal K, Tvedt CR (2020). Elements in scenario-based simulation associated with nursing students’ self-confidence and satisfaction: a cross-sectional study. Nurs Open.

[12] Cogo ALP, Pai DD, Aliti GB, Hoefel HK, Azzolin KO, Busin L (2016). Case studies and role play: Learning strategies in nursing. Rev Bras Enferm.

[13] Kim E (2017). The structural relationship between team intrinsic motivation, knowledge sharing, and team efficacy for college students. Korea Assoc Yeolin Educ.

[14] Gully SM, Incalcaterra KA, Joshi A, Beauien JM (2002). A meta-analysis of team-efficacy, potency, and performance: interdependence and level of analysis as moderators of observed relationships. J Appl Psychol.

[15] Lee H, Lee H (2016). Effects of systems thinking on high school students’ science self-efficacy. J Korean Earth Sci Soc.

[16] Stalter AM, Mota A (2018). Using systems thinking to envision quality and safety in healthcare. Nurs Manag.

[17] Yune SY, Lee EY, Park KH. Relationship between communication, interpersonal understanding, proactivity in problem solving and team efficacy medical students and nursing students. AJMAHS. 2018. 10.21742/AJMAHS.2018.02.37.

[18] Kang S, Lim Y (2014). Development and application of integrated nursing practice program preceded role-play related to clinical communication situation. J Korea Acad-Ind Coop Soc.

[19] Mikkelsen J, Reime MH, Harris AK (2008). Nursing students’ learning of managing cross-infections–scenario-based simulation training versus study groups. Nurse Educ Today.

[20] Delnavaz S, Hassankhani H, Roshangar F, Dadashzadeh A, Sarbakhsh P, Ghafourifard M (2018). Comparison of scenario based triage education by lecture and role playing on knowledge and practice of nursing students. Nurse Educ Today.

[21] Abedini Z, Parvizy S (2019). Student’s perceptions of using scenario-based education to improve civility: a mixed method study. J Adv Med Educ Prof.

[22] Jeong HJ (2015). The development of compensated learning program using role-playing and measurement of learning outcomes on maternity nursing practical education for nursing students. J Korea Entertain Ind Assoc.

[23] Marshall LC. The relationship between* efficacy, teamwork, * effort and patient satisfaction. Los Angeles: University of Southern California; 2003.

[24] Kwon EM. The correlation among team efficacy, interpersonal understanding, proactivity in problem solving and team performance. Unpublished master thesis. Graduate school of education. Seoul: Ewha Woman’s University; 2010.

[25] Lee H, Kwon H, Park K, Lee H (2013). An instrument development and validation for measuring high school students’ systems thinking. J Korean Assoc Res Sci Educ.

[26] Hong CM (2018). The effects of simulation on nursing students’ clinical competence, communication skills, and team efficacy. AJMAHS.

[27] Kim KH (2017). Team-based blended-learning’s effect on critical thinking disposition, problem-solving, and team-efficacy of female nursing students. Korean J Women Health Nurs.

[28] Cho O, Hwang K. The effects of simulation-based education on nursing students’ presence in education, systems thinking and proactivity in problem solving. J Korean Acad Soc Home Healthc Nurs. 2016. 10.22705/jkashcn.2016.23.2.147.

[29] Im Y, Lee H (2014). Development and analysis of effects of writing educational program for improving system thinking ability. J Learn Cent Curric Instr.

[30] Dolansky MA, Moore SM (2013). Quality and safety education for nurses (QSEN): the key is systems thinking. Online J Issues Nurs.

[31] Stalter AM, Phillips JM, Ruggiero JS, Scardaville DL, Merriam D, Dolansky MA (2017). A concept analysis of systems thinking. Nurs Forum.

[32] Moazez M, Miri S, Foroughameri G, Farokhzadian J (2020). Nurses’ perceptions of systems thinking and safe nursing care: a cross-sectional study. J Nurs Manag.

[33] Fura LA, Wisser KZ (2017). Development and evaluation of a systems thinking education strategy for baccalaureate nursing curriculum: a pilot study. Nurs Educ Perspect.

[34] Byeon DH (2019). The effect of simulation-based integrated clinical practice education on the flow, learning presence and proactivity in problem solving for nursing students. Korean Soc Nurs Res.

[35] Park M, Choi D (2018). The effect of simulation integrated with problem based learning on system thinking, learning flow, proactivity in problem solving and performance ability for medication in nursing students. J Digit Converg.

[36] Yildirim JG, Calt AC, Ardahan M (2019). Problem-solving skills of university nursing students and factors affecting them: a cross-sectional study. J Pak Med Assoc.

[37] Kang M, Chung K, Cho J. A design and effect of design thinking-based team project learning in nursing clinical practice. J Korea Contents Assoc. 2019. 10.5392/JKCA.2019.19.03.336.

